# Neurological Complications of COVID-19: Guillain-Barre Syndrome Following Pfizer COVID-19 Vaccine

**DOI:** 10.7759/cureus.13426

**Published:** 2021-02-18

**Authors:** Sadia Waheed, Angel Bayas, Fawzi Hindi, Zufe Rizvi, Patricio S Espinosa

**Affiliations:** 1 Neurology, Florida Atlantic University Charles E. Schmidt College of Medicine, Boca Raton, USA

**Keywords:** neurologic complications, guillain-barre syndrome (gbs), covid-19 vaccine

## Abstract

Since the first case of severe acute respiratory syndrome coronavirus 2 (SARS-CoV-2) was reported in Wuhan, China, in December 2019, Coronavirus - 19 (COVID-19) has become a global pandemic with multiple neurological complications. In December 2020, two vaccines have been approved in the United States for the prevention of COVID-19. We report a case of Guillain-Barre Syndrome (GBS) after receiving the first dose of Pfizer - COVID-19 vaccine.

## Introduction

The first case of severe acute respiratory syndrome coronavirus 2 (SARS-CoV-2) was reported in Wuhan, China, in December 2019 [1], and in few weeks became a global pandemic. Coronavirus - 19 (COVID-19) predominantly causes respiratory illness with symptoms ranging from mild illness presenting with myalgia, sore throat, cough, fever, anosmia, and diarrhea [1], to more moderate-to-severe symptoms of acute respiratory distress syndrome, multi-organ failure and eventually death. Multiple neurological complications have been associated with COVID-19 infection [2,3]. In December 2020, the FDA approved two vaccines for the prevention of COVID-19 infection. In the clinical trials of the vaccine, multiple side effects have been reported ranging from mild symptoms including but not limited to injection site pain, myalgia, fatigue, and fever to more serious side effects including anaphylactic shock [4,5]. However, Guillain-Barre Syndrome (GBS) after receiving COVID-19 vaccine has not been reported to date (February 2, 2021) to the best of our knowledge. We report the first reported case of GBS after receiving the first dose of Pfizer COVID-19 vaccine.

## Case presentation

**Figure 1:**
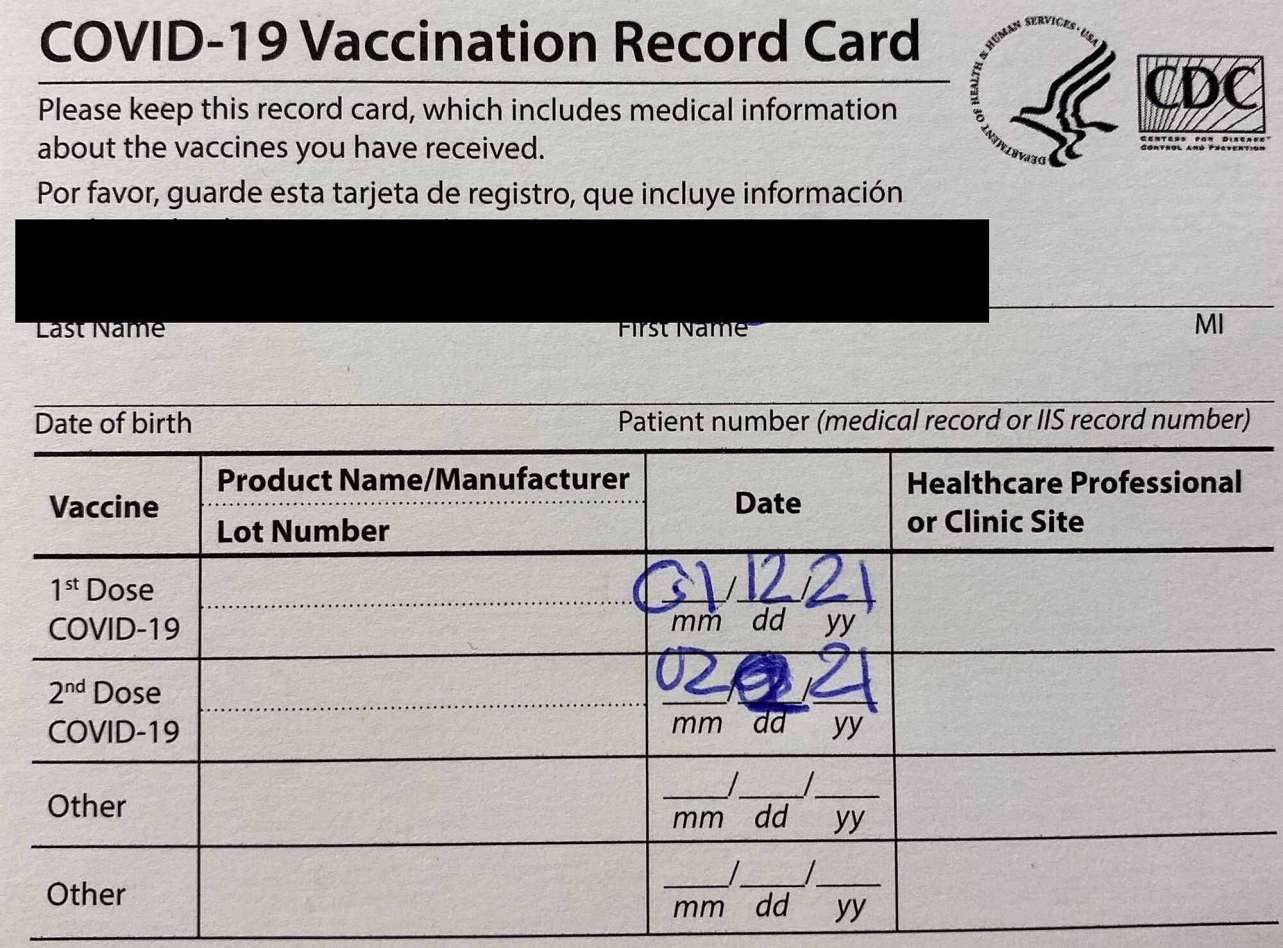
Patient’s vaccination record.

**Figure 2:**
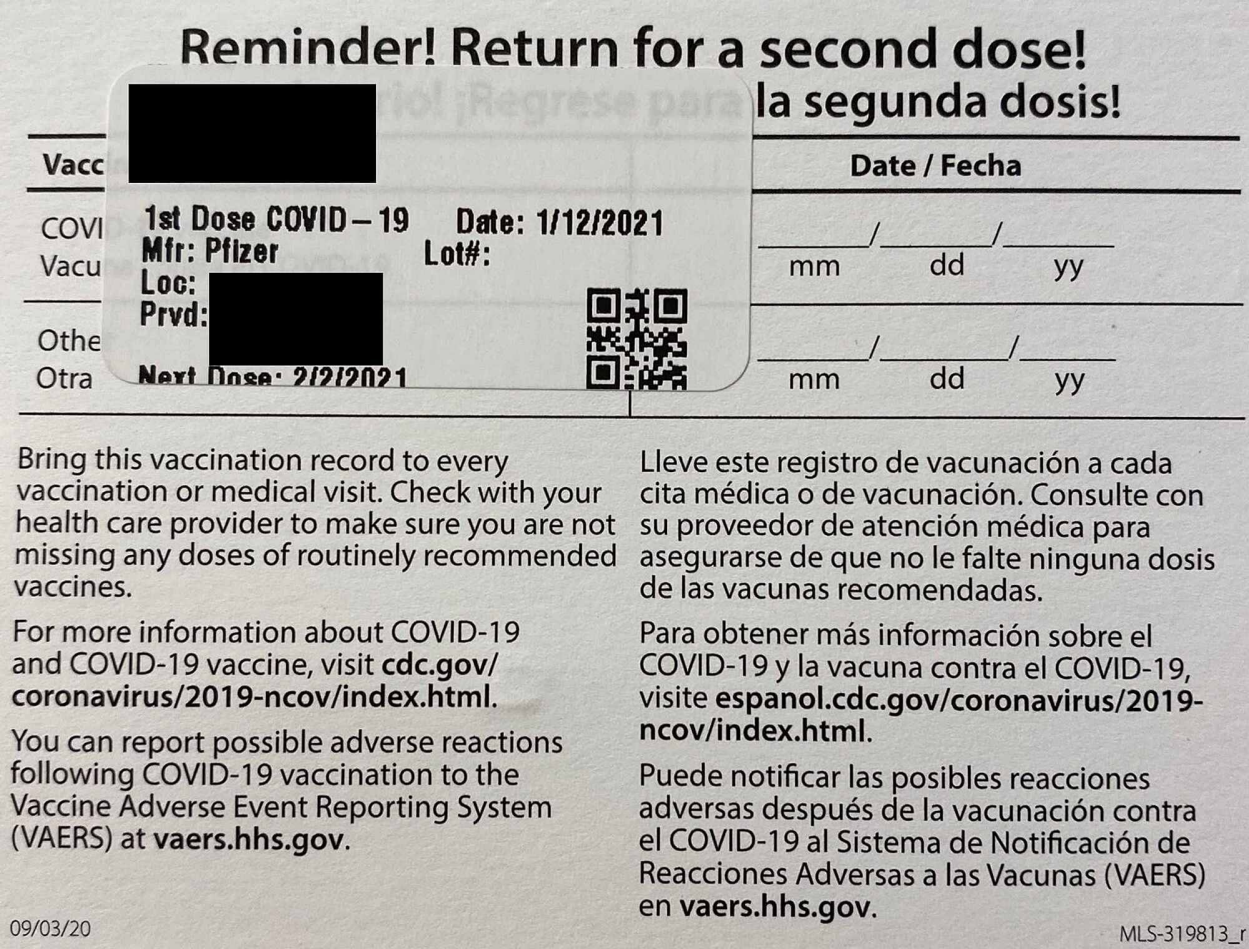
Patient’s vaccination record.

**Figure 3:**
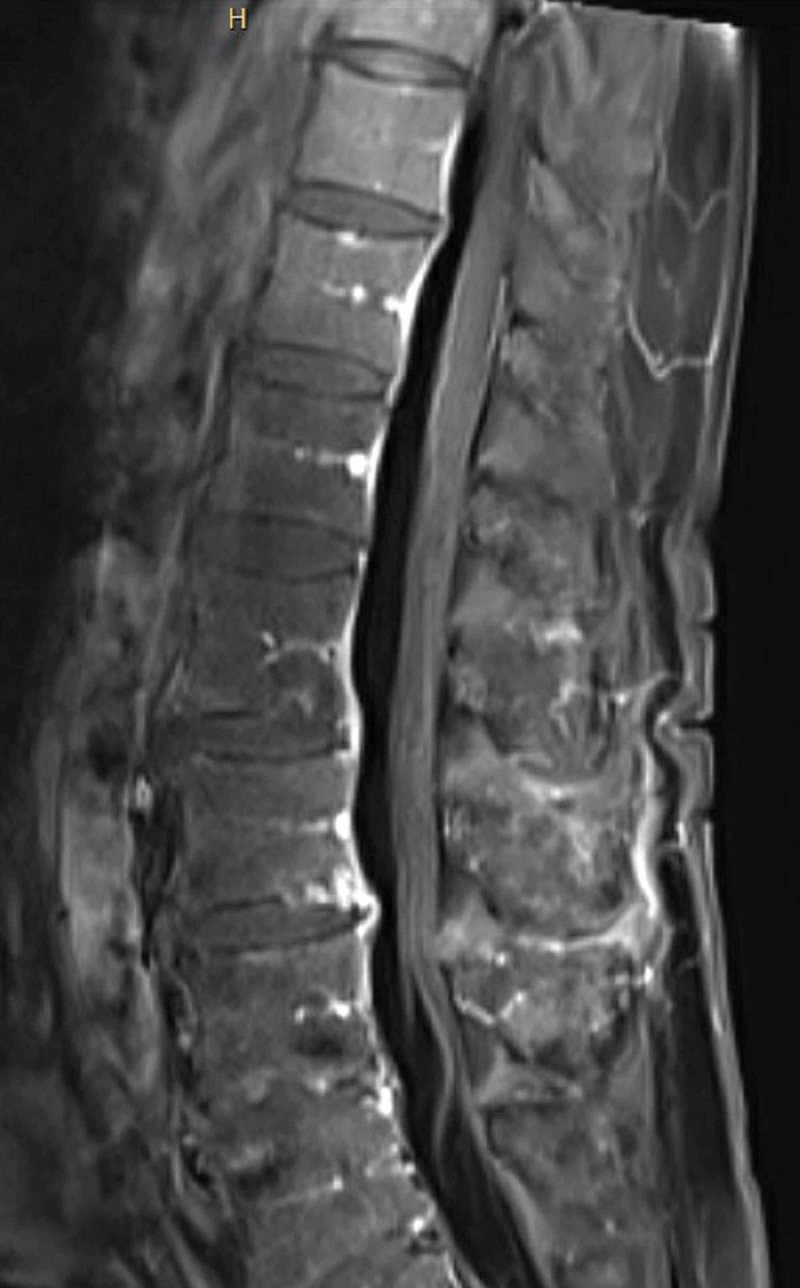
MRI lumbar spine with contrast Sagittal T1 post-contrast MRI lumbar spine demonstrating enhancement of cauda equina nerve roots indicative of Guillain-Barre Syndrome

An 82-year-old highly functional female at baseline without significant comorbidities presented to the emergency department with generalized body aches, paresthesia, and difficulty walking. She received her first dose of the Pfizer COVID vaccine two weeks prior to presentation (see Figures 1, 2). The patient reported generalized malaise and body aches during the first week after receiving her vaccination. However, during the second week, she had worsening symptoms and noticed increased difficulty in walking to the point where she had to use a walker for ambulation. The patient then subsequently sustained a fall due to her weakness, which prompted her visit to the emergency room. Her physical examination on presentation revealed normal mental status and speech. She had an unremarkable cranial nerve examination and no visible facial weakness or asymmetry was appreciated. Motor examination demonstrated normal bulk and tone in bilateral upper and lower extremities, strength in bilateral upper extremities was noted to be 5/5 in both proximal and distal muscles, she had full extension and flexion of her wrists. Although she was able to sustain her bilateral lower extremities against gravity for over five seconds, the examination of muscle group strength testing showed muscle weakness of 4/5 in hip flexors. Her sensation to light touch was intact in bilateral upper and lower extremities but decreased to pinprick in bilateral lower extremities up to the knees. No dysmetria was noted on finger to nose testing. The patient had areflexia in both upper and lower extremities. CT of the brain was normal. Routine labs were unrevealing. Coronavirus Cov-2 PCR was negative. A lumbar puncture was performed and cerebrospinal fluid analysis showed albuminocytologic dissociation (protein of 88 and WBC of 4), consistent with the diagnosis of GBS. The patient was admitted to the Neurology unit and started on intravenous immunoglobulin (IVIG). The patient developed labile blood pressure on day 2 that resolve before discharge. She developed back pain and MRI lumbar spine demonstrated the enhancement of cauda equina nerve roots also consistent with the diagnosis of GBS (see Figure 3). The patient did not show any signs of respiratory compromise and clinical improvement was appreciated after three doses of IVIG, the patient completed five days of IVIG. No complications were observed during or after the treatment. The patient received physical therapy during the hospital stay and was discharged to acute rehabilitation facility. She was referred to the outpatient Neurology Clinic for follow up Nerve Conduction Study (NCS) and electromyography (EMG) to be done in three to four weeks. The Centers for Disease Control (CDC) was notified about this complication associated with the vaccination.

## Discussion

GBS is an inflammatory polyradiculoneuropathy associated with numerous viral infections [6]. Approximately one-third of patients with GBS can develop respiratory failure requiring respiratory support and admission to the intensive care unit (ICU) for intubation [7]. GBS is one of the few leading causes of acute flaccid paralysis in developed countries and can present with varying degrees of weakness, sensory abnormalities, and autonomic dysfunction. Even though the exact pathophysiology is still unknown, it is believed that an autoimmune response plays a role in the pathogenesis of this disease [8].

Since the pandemic outbreak and constantly increasing numbers of COVID-19 respiratory illness caused by SARS-CoV-2, worldwide mass vaccination campaigns have taken place in order to control the pandemic [6,7]. Understanding the epidemiology of the disease and the adverse events arising because of the vaccination is crucial. Failure to appreciate these issues can lead to unnecessary morbidity and mortality [7]. The COVID-19 vaccines approved by the FDA are synthetic messenger RNA vaccines also called mRNA vaccines. Inside the human body, mRNA enters the human cell and instructs the cells to identify the spike protein found on the surface of SARS-COV-2, the virus that causes COVID-19. Our bodies then recognize the spike protein as an invader and produce antibodies against it. Later, if these antibodies encounter the actual virus, they are ready to recognize and kill the virus before it can cause illness. In some patients, this immune response can trigger autoimmune processes that lead to the production of antibodies against the myelin and cause GBS.

## Conclusions

We report the first case of COVID-19 post vaccine associated GBS. In this pandemic and with ongoing worldwide mass vaccination campaign, it is critically important for clinicians to rapidly recognize neurological complications or other side effects associated with COVID-19 vaccination. We would like to highlight that the risk of neurological complications or any other adverse effect associated with COVID-19 vaccination is low and the benefits of the vaccination outweigh any potential risks or side effects, both at the individual and society levels. We encourage and support the recommendations of the CDC and WHO guidelines for COVID-19 vaccination.
